# Making Activity Recognition Robust against Deceptive Behavior

**DOI:** 10.1371/journal.pone.0144795

**Published:** 2015-12-11

**Authors:** Sohrab Saeb, Konrad Körding, David C. Mohr

**Affiliations:** 1 Center for Behavioral Intervention Technologies, Department of Preventive Medicine, Northwestern University, Chicago, Illinois, United States of America; 2 Rehabilitation Institute of Chicago, Department of Rehabilitation and Physical Medicine, Northwestern University, Chicago, Illinois, United States of America; Ulm University, GERMANY

## Abstract

Healthcare services increasingly use the activity recognition technology to track the daily activities of individuals. In some cases, this is used to provide incentives. For example, some health insurance companies offer discount to customers who are physically active, based on the data collected from their activity tracking devices. Therefore, there is an increasing motivation for individuals to cheat, by making activity trackers detect activities that increase their benefits rather than the ones they actually do. In this study, we used a novel method to make activity recognition robust against deceptive behavior. We asked 14 subjects to attempt to trick our smartphone-based activity classifier by making it detect an activity other than the one they actually performed, for example by shaking the phone while seated to make the classifier detect walking. If they succeeded, we used their motion data to retrain the classifier, and asked them to try to trick it again. The experiment ended when subjects could no longer cheat. We found that some subjects were not able to trick the classifier at all, while others required five rounds of retraining. While classifiers trained on normal activity data predicted true activity with ~38% accuracy, training on the data gathered during the deceptive behavior increased their accuracy to ~84%. We conclude that learning the deceptive behavior of one individual helps to detect the deceptive behavior of others. Thus, we can make current activity recognition robust to deception by including deceptive activity data from a few individuals.

## Introduction

Human activity recognition based on the data captured from accelerometers, video cameras, and custom-designed sensors has been studied since the 1980s [1–6]. Some devices, such as the Microsoft Kinect, are specifically designed to collect data that can easily be used to infer the physical activities of a person [7], however, these devices are neither portable nor ubiquitous. Smartphones and wearable activity trackers, on the other hand, offer both portability and popularity. These devices usually come with inertial measurement units (IMUs) that can be used to detect the physical activities of their users: whether they are sedentary, walking, running, sitting in a car, etc. [8], regardless of where they carry their device [9]. Thus, smartphones and wearable devices seem ideal platforms for daily-life monitoring of an individual’s physical activities.

Healthcare providers use mobile and wearable sensor-based activity recognition for a variety of purposes. A number of health insurance companies collect activity data from their customers to adjust their risk estimates and costs according to each individual’s daily-life behaviors. For example, one life insurance company offers 15% discount if their customers are physically active for at least 15 minutes per day [10]. In other healthcare areas, such as mental health and physical rehabilitation, researchers and clinicians are interested in monitoring the physical activities of their patients in their daily life. Researchers might want to detect symptoms of certain disorders and their severity using the activity data recorded from their patients [11, 12]. Clinicians, on the other hand, might be interested in finding out whether or not their patients follow their advice in doing and/or refraining from certain activities that supposedly improves the outcome of the treatment [13, 14].

Whether the goal is to estimate insurance costs or to monitor patients, it is crucial for activity tracking systems to be reliable. One source of unreliability can be inaccuracy of the sensors or the failure of the algorithms, which is usually quantified in activity tracking algorithm development. A second source, which is currently ignored, is the users’ behavior. In fact, users may deliberately trick activity recognition systems into detecting activities that are different from the ones they actually perform. In the health insurance example, users may try to show that they are more active and thereby receive a discount. All they need to do is to shake the device while they sit on their couch. In a patient monitoring scenario where patients are monitored by an activity tracker to ensure they do not move out of bed without asking for assistance, they might find a way of walking which the activity tracker cannot detect. Therefore, as healthcare providers rely more on activity trackers, there is an imminent need to make these systems robust against deceptive behavior.

Very few studies have tried to make activity recognition robust against cheating. A first attempt was to train a neural network classifier to distinguish between the accelerometer data generated by normal walking from the ones generated by shaking the activity tracker device, however no cross-validation was performed to measure the accuracy of the method [15]. In a study of the cheating behavior in a soccer video game, researchers used the intensity of motions as an additional feature in order to prevent gamers from cheating by mimicking the actual activity with low effort in a smaller scale [16]. Finally, a more recent study asked subjects to trick a smartphone-based activity tracker into detecting walking activity while they were seated, by shaking their phones [17]. This study was able to provide good accuracy on distinguishing between deceptive and normal activities. However, it did not examine whether or not the subjects were still able to cheat after the classifier was trained on the cheating data.

In this study, we asked subjects to deliberately make our smartphone-based activity classifier fail in two distinct settings: Detect ‘walking’ while they were sitting on a chair, and detect ‘sitting’ while they were walking. If they succeeded in cheating, we retrained the activity classifier by incorporating fake activity data into its training dataset, and asked the subjects to try to cheat again. We continued this procedure until the subjects could no longer cheat. To encourage subjects to try their best to trick the classifier, we provided them with a monetary reward each time they succeeded in cheating for an entire round. Finally, we investigated whether training a classifier on the deceptive data from one or a few subjects could make it robust against the deceptive behavior of other subjects.

## Experiment

### Participants

We recruited 14 healthy subjects (5 males and 9 females) within the age range of 23 to 38 years old using advertisements in a Northwestern University newsletter. Subjects were eligible for the experiment if they were able to speak and read English and lived in the Chicago area. Before the experiment, written informed consent was obtained from each subject. The study protocol and the consent procedure was approved by the Northwestern University Institutional Review Board.

### Activity Trials

**Fig 1:**
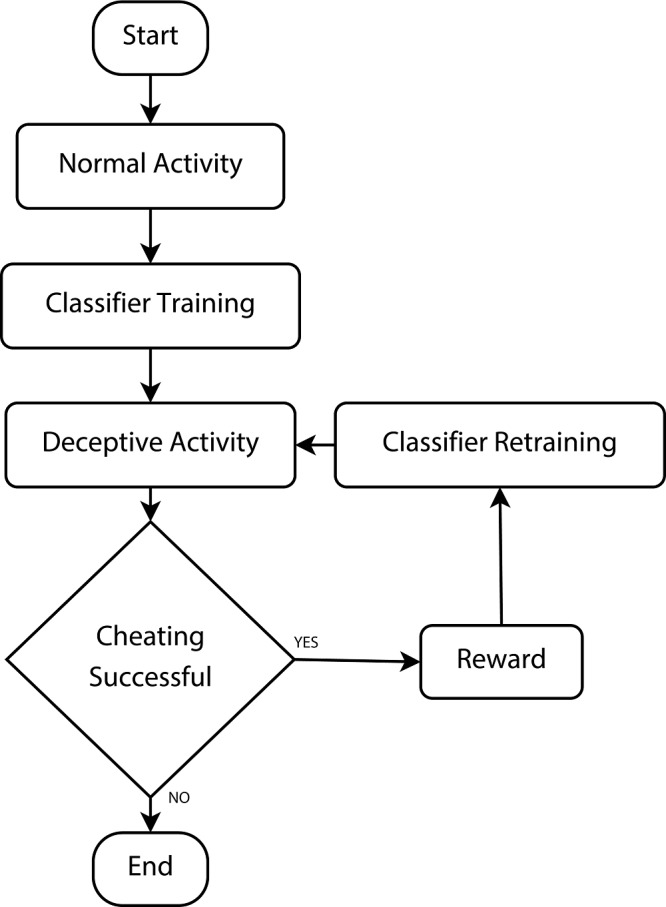
Experiment design overview. Each trial consisted of two types of activities: walking and sitting.

The experiment design is summarized in Fig 1. At the beginning of the experiment, we handed each subject a Google Nexus 5 smartphone running our activity classifier application (see Mobile Application). Then, they went through a number of activity trials while carrying the phone. There were two sets of trials:

1. **Normal Activity Trials.** At the beginning of the experiment, we asked subjects to do one session of sitting and another session of walking, and perform these activities in the way they normally do. Each session lasted for 5 minutes, and subjects were allowed to carry the phone in any way they preferred. We used the collected sensor data to train the classifier application to distinguish between walking and sitting.
2. **Deceptive Activity Trials.** After normal activity trials, we asked subjects to go through a second set of walking and sitting trials, but this time try to trick the classifier: when sitting, make it detect ‘walking’, and when walking, make it detect ‘sitting’. To provide feedback to subjects about the output of the classifier, the mobile app made a beep sound every time it was able to detect the true activity. Subjects were allowed to do anything as long as they remained seated in the sitting trials and continued walking in the walking trials. To encourage them to try their best to trick the classifier, we paid them a $2 reward each time they succeeded in any of the sitting or walking trials, however we did not mention what specific movements to do in order to cheat. If a subject succeeded, we used the collected sensor data to retrain the classifier application, and started the next round of deceptive activity trials after a short break.

#### Ending Criterion

The experiment ended when a subject was not successful in a deceptive activity trial, or the maximum allowed number of trials, 10, was reached. Subjects’ success in each deceptive activity trial was measured by *success rate*, defined as the proportion of the number of evaluations in which they were able to deceive the classifier. If the success rate was greater than 50%, the trial was considered successful, and otherwise, failed. Evaluations were performed once every second, and the trial continued for 5 minutes, equivalent to 300 evaluations, or until the number of either of the detected classes reached 150, whichever came first.

## Mobile Application

We developed an Android mobile application and installed it on the smartphone that the subjects carried during the experiment. The application collected data from accelerometer and gyroscope sensors, extracted their features, ran an activity classifier on the features, and provided feedback to the subject about the output of the classifier by making a beep sound each time the true activity was detected. A copy of the sensor data was also saved and transmitted to a desktop computer to retrain the classifier after each trial.

The application consisted of three modules: Data acquisition, feature extraction, and activity classifier. In the following, we will explain each of these modules.

### Data Acquisition

The data acquisition module captured motion data using the accelerometer and gyroscope sensors on the device. The accelerometer measured the acceleration along the phone’s X, Y, and Z axes, and the gyroscope measured the rotational velocity around each of these axes. These two sensors complement each other, and allow for the separation of the Earth gravity component of the signals. In fact, a recent study showed that maximum accuracy in activity recognition can be achieved by combining these two [18].

The accelerometer and gyroscope sensors on a Nexus 5 device can be set to sample at different frequencies, from 15 Hz to 200 Hz. Here, we set the sampling frequency for both sensors to 50 Hz, which has been shown to be sufficient for capturing the dynamics of human motion [8].

### Feature Extraction

The feature extraction module calculated various features of the motion sensor data, which captured the patterns of change in the accelerometer and gyroscope sensor data. In fact, one cannot directly use raw sensor data for activity classification as there is a new sample arriving every 20 milliseconds. Thus, we extracted features from the raw sensor data and then used these features to perform activity classification.

To compute features from the raw sensor data, we divided each of the accelerometer and gyroscope sensor data into frames. Each frame was 200 samples long, corresponding to 4 seconds of data. The frames also had 75% overlap, meaning that adjacent frames had 150 samples, equivalent to 3 seconds, of sensor data in common. Then, we defined and extracted a variety of features from each frame, as listed in Table 1. We had previously used this feature set in a study of activity recognition in Parkinson’s disease patients [19]. The features included descriptive statistics, the magnitude of the second to the 20th Fourier transform components, and cross-correlations between the axes of each sensor. In total, we extracted 260 features from each frame of the accelerometer and gyroscope sensor data.

**Table 1:** The feature set used for activity recognition. Each feature was extracted from both accelerometer and gyroscope sensor data frames.

Feature Set (Accelerometer/Gyroscope)	No. features
Mean, abs mean, RMS	9
Moments (standard deviation, skewness, kurtosis)	9
Moments (mean, standard deviation, skewness, kurtosis) of the derivative	12
Extremes (min, max, abs min, abs max)	12
Histogram (counts for -3 to 3 z-score bins)	18
Fourier coefficients (2nd to 20th coefficients)	57
Overall mean	1
Cross-correlations (XY, YZ, ZX) mean and abs mean	6
Angular cross-correlations (XY, YZ, ZX) mean and abs mean	6
**Features from each sensor**	**130**
**All (Accelerometer + Gyroscope)**	**260**

### Activity Classifier

We used random forests [20] as the activity classifier in our mobile application. A random forest is an ensemble of decision trees, with each providing a prediction, or *vote*, about the class the input data belongs to. The forest’s prediction is determined by averaging over the predictions of individual trees. Each tree in a random forest only sees a subset of features and a subset of input data samples, which makes the forest less prone to overfitting and a better candidate for generalization to unseen data [21]. This was the main reason why we chose random forests as activity classifiers.

The random forest classifier used the features extracted from motion sensors to distinguish between sitting and walking activities. It consisted of 200 decision trees. The training dataset for each tree was created by randomly and uniformly sampling from the forest’s training set. In addition, each decision split in a tree randomly sampled 16 out of 260 features, and used a Fisher information gain criterion to determine the best feature out of 16, and the decision boundary. The forest’s predicted class was the class gathering most of the trees’ votes, and its confidence was the proportion of the number of trees voting for the winning class. The activity classifier module was optimized such that it provided the classification results in less than 1 second, so that feedback was provided to the subject in time.

## Classifier Training and Evaluation

### Personal Classifiers

In the beginning, we trained a classifier that could distinguish between normal sitting and walking activities of each subject. After the subject finished a deceptive activity trial, we retrained the activity classifier using the sensor data collected from that last trial. Since this was a computationally demanding process, we used a desktop computer. First, we transferred the new accelerometer and gyroscope data to the computer. Then, we extracted features from these data and retrained the classifier using MATLAB. Finally, we transferred the trained classifier back to the phone using a JavaScript Object Notation (JSON) format. This classifier was then used by the mobile application in the next trial.

### Global Classifiers

To understand how learning the cheating behavior of an individual could be generalized to other individuals, after finishing the experiment on all subjects, we trained another set of classifiers on data from multiple subjects and cross-validated them on the subjects that were not used in training. We varied the number of training subjects from 1 to 13. This procedure was repeated 10 times with different training and test samples each time.

We trained two sets of classifiers. One set was trained only on normal activity data which we had obtained at the beginning of the experiments. We call these *baseline* classifiers. The other set was trained on both normal and deceptive activity data, which we call *expert* classifiers. To make sure that the comparison between the baseline and the expert classifiers was fair, and that the expert classifiers did not benefit from having larger number of samples in their training set, we created bootstrapped training sets such that the number of training data points for both expert and baseline classifiers was equal.

We measured each classifier’s performance by measuring its accuracy, sensitivity, and specificity in detecting the true activity.

## Results

We asked subjects to try to trick our activity classifier mobile application by making it detect ‘walking’ while they were seated and ‘sitting’ while they were walking. When they succeeded in cheating, we used their motion data to retrain the classifier, and asked them to try to trick it again. We first wanted to see if it was harder for a subject to trick an activity classifier that had been trained on deceptive activity data. The second question was if training classifiers on one or multiple subjects’ deceptive activity enabled them to become more robust against the deceptive activity of other subjects. The data behind visualizations in this section are available in S1 Data.

### Participants’ Success in Deceiving Classifiers

First, we quantified how well subjects could cheat over the course of the experiment. Four out of 14 subjects were not able to cheat at all from the beginning. The rest of the subjects succeeded in between 1 and 3 retraining rounds. None of the subjects were able to reach the maximum number of retraining rounds (10). Thus, most subjects were able to deceive the classifier to some degree.

**Fig 2:**
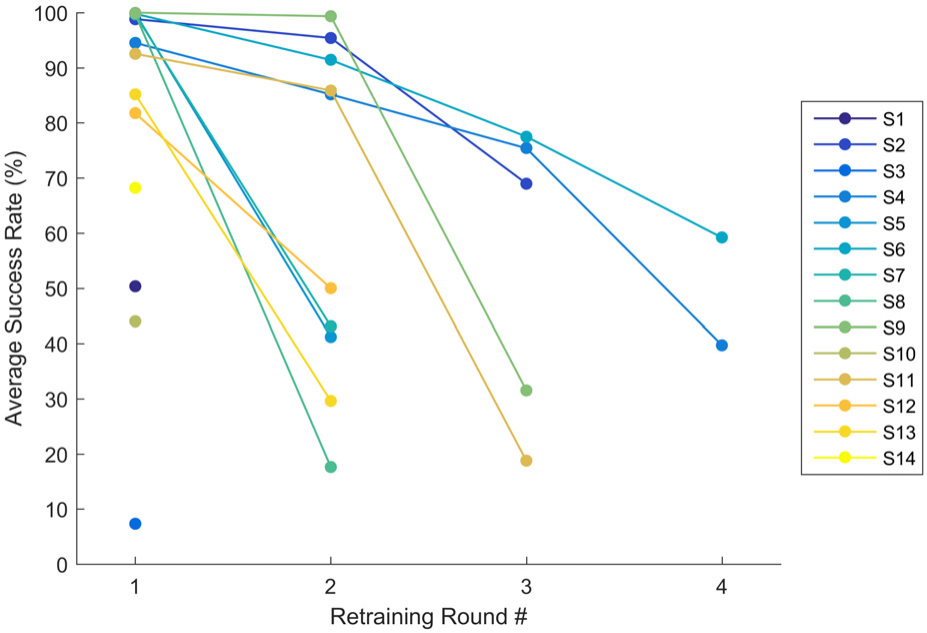
Average success rate of subjects in walking and sitting trials, indicating how successful they are at cheating. Each colored dot represents a subject’s success rate at a trial. The dots which are not connected to any line belong to subjects who failed in the first deceptive activity trial.

If the approach is successful, then cheating must get harder over subsequent rounds of the retraining iterations. Fig 2 shows the success rate (see Activity Trials) of each subject at each round averaged over walking and sitting trials. Indeed, most subjects were very successful in the first cheating trial, with a success rate of above 95%, which gradually fell after each retraining. Clearly, the classifier got better over the course of the experiment, making it harder for subjects to cheat.

Subjects used a variety of cheating strategies. For the fake walking trials, where they were actually sitting on a chair, most subjects shook the phone with their hands as a strategy to trick the classifier. Some subjects tried to put the phone in their pocket and move their torso or legs to induce sensor readings that were similar to a true walk. When shaking their phones, subjects also used different strategies, such as moving the phone linearly back and forth, drawing circles, imitating the hand movements of a true walk, and rotating the phone. For the fake sitting trials where they were walking, most subjects tried to walk very smoothly, but some also tried to walk with a pace different from their normal walking trial. Others tried to move the phone with their hand in such a way that the overall impact on the phone was reduced.

**Fig 3:**
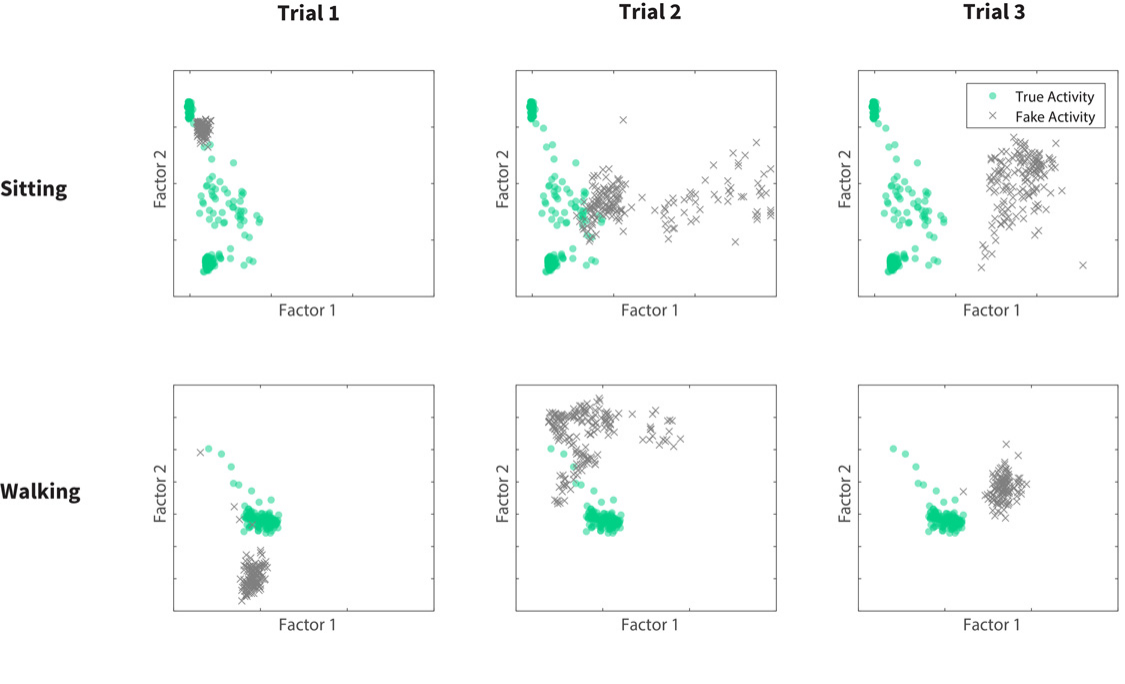
2-dimensional representation of feature values collected from a single subject who succeeded in 3 trials. We used a 2-component factor analysis. Green circles show normal activity and grey crosses indicate fake activity factors. The changing location of fake activity data points across trials shows different strategies taken by the subject.

The cheating strategies used by subjects are reflected in a low-dimensional representation of their motion features depicted in Fig 3. In each plot, the green circles correspond to the baseline (non-deceptive) sensor data for either sitting (top plots) or walking (bottom plots), and the grey crosses are deceptive behavior data. It is evident from this figure that the subject’s strategy in cheating, which is manifested in the location of grey dots, changes from trial to trial. Therefore, our experiment successfully motivated subjects to try various strategies to trick the activity classifier.

### Machine’s Success in Detecting True Activities

**Fig 4:**
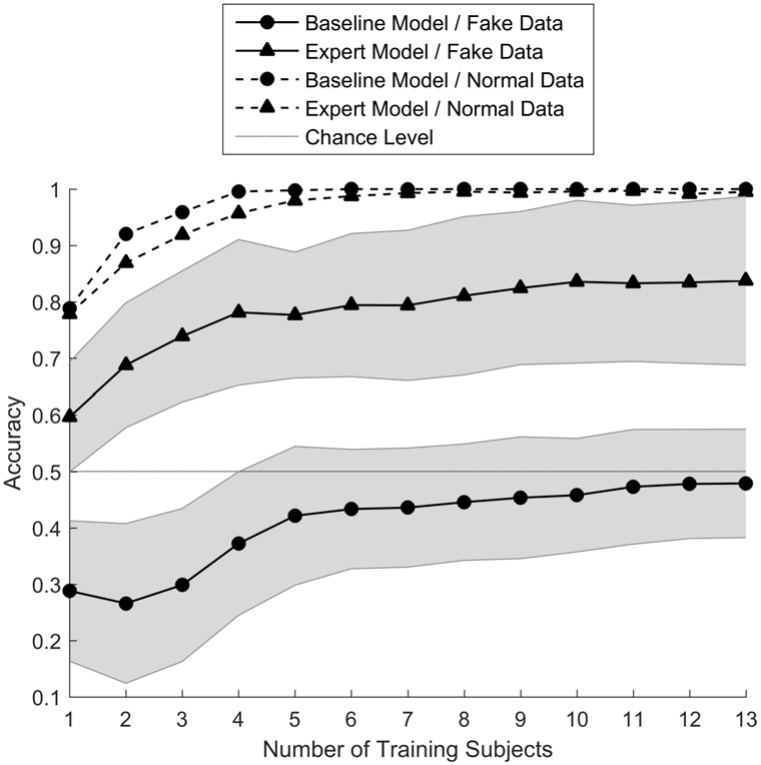
Accuracy of the baseline (circles) and the expert (triangles) models in classifying deceptive (solid) and normal (dashed) data, as a function of the number of training subjects. Dotted line shows the chance level (50%) and the shaded areas show standard deviation of the accuracies.

We evaluated the performance of baseline and expert classifiers in detecting the true activities, by varying the number of training subjects from 1 to 13 and testing them on subjects not present in training sets. The single-subject classifiers were the ones that were trained during the experiment and used in the mobile application, while the rest of the classifiers were trained after data from all subjects was collected. Fig 4 shows how expert classifiers outperformed baseline classifiers in detecting fake activity for any number of training subjects. Nevertheless, the accuracy of both classifiers increased by increasing the number of training subjects.

In the leave-one-subject-in (LOSI) case (far left), where the classifiers were trained on one subject and tested on the rest, the baseline classifier had an accuracy of ~38% while the expert classifier’s accuracy was ~59%. For the leave-one-subject-out (LOSO) case (far right), where the classifier was trained on all subjects except one and tested on the remaining subject, these numbers were ~47% and ~84%, respectively. Thus, the accuracy of the baseline classifier did not get better than chance (50%) by increasing the number of training subjects, while the expert classifier had good accuracy in the LOSO cross-validation setting.

We also evaluated how the classifiers performed on normal activity data, before and after training on deceptive activity. These are shown by dashed lines in Fig 4. We found that the accuracy of both baseline and expert classifiers increased by including more training subjects, nevertheless their performances did not considerably differ. Therefore, training the classifiers on deceptive activity data did not affect their performance on normal activity data.

Next, we wanted to see if the performance of the classifiers was different for walking and sitting activities. Table 2 shows the precision and the recall of the expert classifier in detecting each of the activity classes, in the LOSI and LOSO settings. Interestingly, there is a considerable difference in recall between the two classes, implying that most of the inaccuracy of the LOSI classifier was caused by inaccuracy in detecting the ‘walking’ activity. In other words, the LOSI model was more vulnerable to deceptive walking. This difference disappeared when more training subjects were used, in the LOSO classifier. Thus, in the end, the expert classifier with sufficient number of training subjects was able to perform well in both walking and sitting classes.

**Table 2:** Precision, recall, and accuracy of the expert model in detecting the true activity.

	Leave-One-Subject-In		Leave-One-Subject-Out	
	Precision	Recall	Precision	Recall
**Sitting**	55.1%	75.8%	83.4%	75.2%
**Walking**	78.2%	41.3%	79.3%	88.8%
**Total Accuracy**	**59.4%**		**83.8%**	

**Fig 5:**
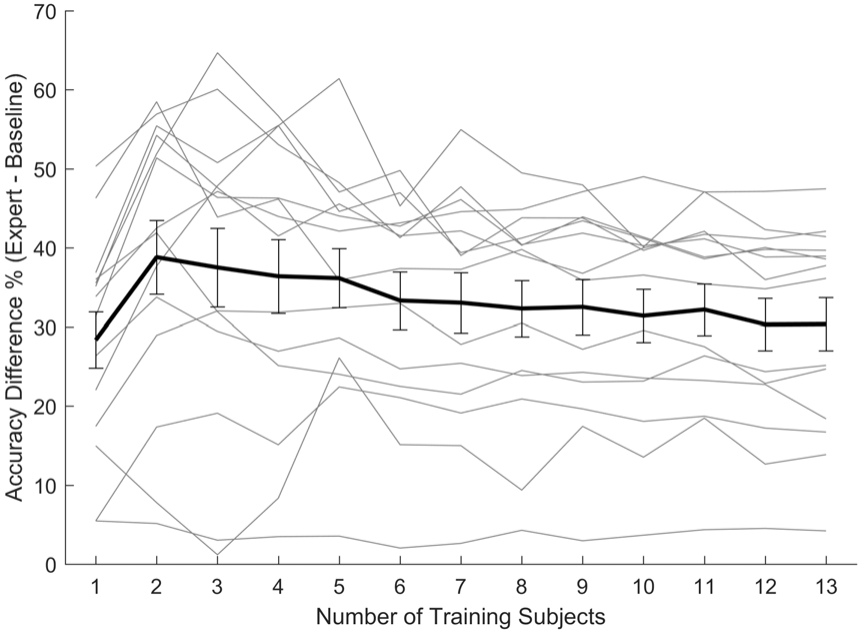
Difference (%) between the accuracy of the expert and the baseline classifiers, when varying number of training subjects is used. Each gray line shows the accuracy difference when one subject’s deceptive data is used as test, and the black line shows the average and its standard deviation as error bars. In all tests the expert classifiers perform better than the baseline classifiers (the difference is positive). While the relationship between the accuracies and the number of training subjects is noisy, it does not vary much for each test dataset.

Finally, we evaluated how the gap between baseline and expert classifiers varied depending on which subject was used for test. Fig 5 shows the difference between the accuracy of the baseline and the expert classifiers as a function of the number of training subjects. Each gray line shows this difference when one subject is used as test, and the black line shows the average for all test subjects. It seems that the average difference is maximized when the number of training subjects is 2, although the overall variation is negligible. However, this difference considerably varies across the test subjects. Therefore, the gap between the baseline and the expert classifiers’ performance depends on which subject is used for test, but it does not depend on the number of training subjects.

## Discussion

Activity tracking systems are vulnerable to deceptive behavior. We showed that training activity classifiers on deceptive activity data from a few subjects enabled these classifiers to detect the deceptive activity of other subjects with good accuracy. The performance of the classifiers trained using both normal and deceptive activity data was considerably and consistently better than the ones trained only using normal activity data. Therefore, it seems that machine learning of deceptive behavior can be generalized across individuals. An implication of this result is that including deceptive activity data in the training of current activity tracking systems might substantially increase their performance in handling deceptive behavior.

Tricking an activity classifier which is only trained on normal activity data is simple. This was evident in our experiments, where most subjects were able to cheat in the first trial with a success rate of close to 100%. However, the deceptive activity data collected in this way will not have enough variability within and across the subjects. Our experiment was able to generate more variability by retraining the classifier on the deceptive data collected from each trial, and asking the subjects to try to cheat again. This made subjects use a new cheating strategy for each trial, resulting in a rich deceptive activity dataset.

Nevertheless, this study has a number of limitations that must be considered. First, we did not explore many other ways of cheating in activity recognition. For example, we did not account for impersonation, which means handing the mobile phone to another person to do activities. This type of cheating was explored in a recent study [17]. Furthermore, there can be other ways for individuals to cheat that were not possible in our experiment setting. For instance, one might put their phone in a clothes dryer or stick it to their pet in order to make it detect “walking”. In fact, we were assuming that subjects were carrying their phone all the time.

Second, we only tried to distinguish between walking and sitting. There are several other classes of physical activity, such as stair climbing, running, or driving a car, that can be detected by some smartphone-based activity tracking services such as Google’s Activity Recognition API on Android devices. When more activity classes are present, it is possible that the cheating behavior causes the confusion to spread across many classes (e.g., confusing walking with driving or sitting with stair-climbing), which makes it more difficult to handle the deceptive behavior. Our goal here was to keep the number of classes low and instead focus on maximizing the diversity of cheating strategies that subjects were using.

Finally, smartphones are less portable than wearable activity trackers which might be better candidates in situations such as intensive sports activities. However, our subjects were carrying the phone all the time, and we limited the phone sensors to accelerometer and gyroscope. This setting minimized the difference between phone-based and wearable-based activity tracking in our experiment, and thus we believe that our methodology can be applied to the wearable sensor technology as well. In fact, a future study can perform the same experiment with wearable activity trackers.

Physical activity tracking is becoming more and more popular in healthcare, as well as other areas. A law firm in Canada has used the activity data collected from their client’s activity tracker device to show the adverse effects of an accident she had in the past [22]. Although this is the first known case of this kind, it is very likely that courts in future incorporate activity data into their evidence gathering procedures. We showed the potential to considerably improve the robustness of current physical activity tracking systems, and making them more reliable by training them on deceptive along with normal activity data.

Our methodology can be extended to other areas of pattern recognition and ubiquitous computing that are susceptible to deceptive behavior. For example, speaker verification technology on smartphones allows us to detect whether a voice command is issued by the owner of the phone or another person. However, the other person may be able to hack the software by imitating the owner’s voice. One way to make speaker verification more robust against such behavior would be to ask individuals to try to trick it into detecting someone else’s voice, and train the classifier on the data collected in this way. Thus, an experimental setup similar to ours can be used to increase the robustness of speaker verification against deceptive behavior.

## Supporting Information

S1 Data Figures and tables data. This file contains the data that is behind Figs 2–5 and Table 2.
(ZIP)
